# Inflammation and oxidative stress processes in induced precocious puberty in rats

**DOI:** 10.1016/j.heliyon.2024.e40962

**Published:** 2024-12-05

**Authors:** Raluca Maria Pop, Luciana Mădălina Gherman, Elena-Mihaela Jianu, Ștefan Horia Roșian, Mădălin Mihai Onofrei, Lavinia Patricia Mocan, Veronica Sanda Chedea, Ioana Corina Bocsan, Dragoș Apostu, Andreea Roxana Todea, Eva Henrietta Dulf, Jeanine Cruceru, Carmen Mihaela Mihu, Alina Elena Pârvu, Anca Dana Buzoianu

**Affiliations:** a Pharmacology, Toxicology and Clinical Pharmacology, Department of Morphofunctional Sciences, "Iuliu Haţieganu" University of Medicine and Pharmacy, Victor Babeș, No 8, 400012, Cluj-Napoca, Romania; b Academy of Romanian Scientists, Ilfov 3, 050044, Bucharest, Romania; c Experimental Centre of "Iuliu Haţieganu" University of Medicine and Pharmacy, Louis Pasteur, No 6, 400349, Cluj-Napoca, Romania; d Histology, Department of Morphofunctional Sciences, "Iuliu Haţieganu" University of Medicine and Pharmacy, Victor Babeș, No 8, 400012, Cluj-Napoca, Romania; e “Niculae Stăncioiu” Heart Institute Cluj-Napoca, 19-21 Calea Moților Street, 400001, Cluj-Napoca, Romania; f Department of Cardiology—Heart Institute, “Iuliu Haţieganu” University of Medicine and Pharmacy Cluj-Napoca, Calea Moților Street No. 19-21, 400001, Cluj-Napoca, Romania; g Research Station for Viticulture and Enology Blaj (SCDVV Blaj), 515400, Blaj, Romania; h Orthopaedics and Traumatology, Department of Surgical Specialities, Iuliu Hatieganu University of Medicine and Pharmacy, Victor Babeș, No 8, 400012, Cluj-Napoca, Romania; i Department of Automation, Faculty of Automation and Computer Science, Technical University of Cluj-Napoca, Memorandumului Street No. 28, 400014, Cluj-Napoca, Romania; j Pathophysiology, Department of Morphofunctional Sciences, Faculty of Medicine, Iuliu Hațieganu University of Medicine and Pharmacy Cluj-Napoca, 400012 Cluj-Napoca, Romania

## Abstract

This study aimed to assess the influence of different types of blue light sources on male and female rats' puberty onset, the morphologic-induced alterations in reproductive organs tissues, the impact on inflammation and oxidative stress markers, anxiety levels, and mathematical modeling for tissue data interpretation. Four groups of sixteen rats each (8 females and 8 males/group) were investigated: three groups were exposed to blue light from mobile phones (MP), computer screens (PC), or LED lamps (LED) versus the control group (CTRL). The rats in the CTRL group had no exposure while the other groups were exposed for 30 days to the blue light of MP, PC, and LED for 16 h per day. Serum levels of cortisol, TNF-α, IL-6, and MMP-2 and MMP-9 ovaries and testis tissue levels were analyzed using the ELISA technique. Total oxidative stress (TOS), nitric oxide (NO), and malondialdehyde (MDA) in serum were determined spectrophotometrically. Histomorphological examination was performed on both male and female genital organs. Rats of both sexes presented significant early onset of puberty secondary to blue light exposure. LED-emitted light significantly increased TNF-α and MMP-9 levels in both sexes. The MP and PC emitted light significantly affected the levels of MMP-2 in both females and males. Levels of TOS and NO were increased by LED, respectively by MP and LED exposure in female rats. The histopathological examination revealed no statistically significant differences in the ovaries and testes of rats across the different groups. Blue light exposure induces precocious puberty, by accelerating sexual maturation, and triggers the overproduction of MMPs that could promote organic alteration through tissue remodeling. Oxidative stress parameters were upregulated only in female rats, while cortisol levels were higher in male rats.

## Graphical abstract

**Figure:**
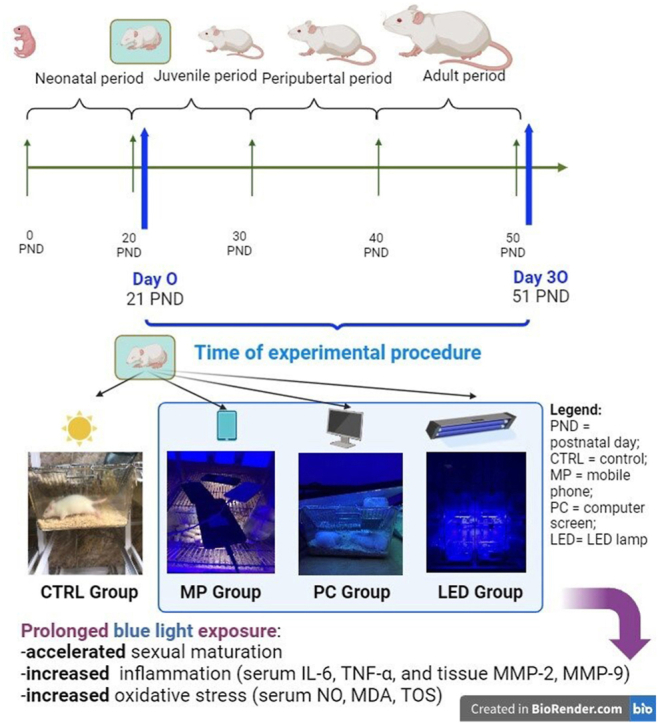

## 1 Introduction

Precocious puberty is defined as the emergence of secondary sexual traits in girls before the age of eight, and in boys before the age of nine [1]. It is classified into two types: central precocious puberty which is gonadotropin-releasing hormone-dependent (GnRH), and peripheral precocious puberty, which is GnRH-independent [1,2]. While conditions such as central nervous system tumors (e.g. hamartomas, astrocytomas) can induce true precocious puberty, the majority of cases are idiopathic [3,4], with a higher incidence in girls than in boys.

Epidemiological studies have shown a rising prevalence of pediatric precocious puberty, influenced by genetic, socioeconomic, and environmental factors, particularly during COVID-19 pandemic [5,6]. Modern technological advancements have significantly altered lifestyles with increased exposure to digital screens emitting artificial blue light, reported before the age of two in children [7,8].

Prolonged blue light exposure emitted from various smart devices affects sleeping patterns, decreases physical activities, and can induce overweight among exposed children [[9], [10], [11]]. Blue light, while part of the natural light spectrum, has been associated with negative effects such as retina structural changes [12], circadian rhythm disturbances [13], impairment in gross motor and language development in children [14], altered sexual maturation, and morphological modifications of the reproductive system [15,16]. Exposure can stimulate the intrinsically photosensitive retinal ganglion cells (ipRGCs) that are responsible for regulating the body's circadian rhythm through modulation of melatonin secretion [17,18]. With a reduction in melatonin levels, the hypothalamus can release more of the GnRH, which triggers puberty onset [19]. Thus, it is believed that there is a significant correlation between blue light exposure and reaching the pubertal stage early [8,20,21]. Also, increased luteinizing hormone (LH) and follicle-stimulating hormone (FSH) levels, characteristic of the central onset of puberty were reported in rats exposed to blue light [8]. Further, disturbances of circadian rhythm after blue light exposure can lead to decreased levels of leptin and increased levels of ghrelin, both linked to increased hunger [22] and consequently increased weight. Thus, precocious puberty is multifactorial, involving environmental factors like light exposure, physical inactivity, obesity, and inflammatory/oxidative stress processes [9,13].

Inflammation, the immune defense mechanism against harmful stimuli [23], mediated by cytokines, chemokines, prostaglandins, leukotrienes, and others [24], affects hypothalamic regulation and inhibits GnRH secretion, potentially delaying [25,26] or promoting puberty [[27], [28], [29], [30]] depending on cytokine levels (e.g., TNF-α, IL-6, and IL-1β). Chronic inflammation is linked to oxidative stress, characterized by excess reactive oxygen species (ROS), with each process exacerbating the other [31,32]. Understanding the role of inflammation and oxidative stress in precocious puberty could lead to preventive strategies.

This study aims to quantify pro-inflammatory cytokines (interleukin-6 (IL-6), tumor necrosis factor-alpha (TNF-α)), metalloproteinases (metalloproteinase-2 (MMP-2), metalloproteinase-9 (MMP-9)), and oxidative stress markers (total oxidant status (TOS), malondialdehyde (MDA), and nitric oxide (NO)) in rats exposed to blue light and assess histological changes in reproductive tissues. Additionally, it examines anxiety via the elevated plus maze test and serum cortisol levels.

## 2 Materials and methods

### 2.1 Chemicals

Alcohol, xylene, and paraffin were purchased from International Laboratory SRL, Cluj-Napoca, Romania. Hematoxylin and eosin were purchased from Sigma Aldrich, Merck Romania SRL, an affiliate of Merck KGaA, Darmstadt, Germany.

### 2.2 Animals

This study enrolled 64 Wistar rats (32 females and 32 males), aged 21 days and weighing on average 40 g. The rats were housed in groups of four with food and water provided ad libitum. The microclimate was maintained at 21–24 °C and 40–45 % humidity. All animal care procedures followed ethical guidelines and were approved by the University of Medicine and Pharmacy “Iuliu Hațieganu” Ethics Committee and by the Sanitary-Veterinary and Food Safety Directorate from Cluj-Napoca (authorization number 380/August 25, 2023).

### 2.3 Experimental design

**Fig. 1:**
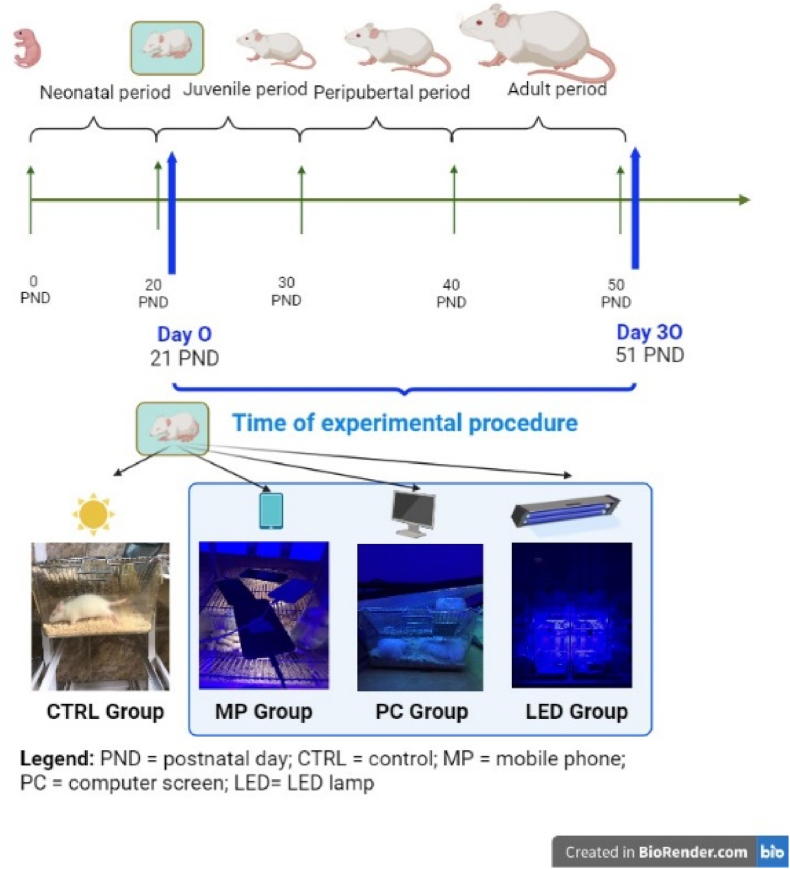
The period of development in male and female rats as related to the time of experimental procedure where the CTRL group was exposed to natural light, the MP group was exposed to the blue light from the mobile phones, the PC group was exposed to the blue light from the computer screen, and LED group was exposed to the blue light from the LED lamp.

Both female (32 animals) and male rats (32 animals) were divided into four groups (n = 8/group). After the random allocation, four distinct groups were formed: a control group (CTRL), a group exposed to blue light from mobile phones (MP), a group exposed to blue light from a computer screen (PC), and a group exposed to blue light from a LED lamp (LED) (Fig. 1). The rats in the control group were maintained under the standard 12-h light/12-h dark cycle. In contrast, the other experimental groups were exposed for 30 days to the blue light of MP, PC, and LED for 16 h per day (from 7:00 a.m. to 11:00 p.m.), with the dark period spanning from 11:00 p.m. to 7:00 a.m. The LED lamp, for experimental use, served as a positive control source of blue light with an irradiance of 450–470 nm, a radiant flux of 753 mW, a radiant efficiency of 75 %, and a photosynthetic photon flux of 2.74 μmol/s. The computer screen LCD Monitor (BenQ GL2250H, 2014) had a resolution of 1920x1080, a refresh rate of 60.00 HZ, and the screen brightness was set at maximum. To prevent any interference caused by the rats' movements inside the cage, the blue light sources were positioned approximately 20 cm away from the animals (Fig. 1).

### 2.4 Timing of puberty

**Fig. 2:**
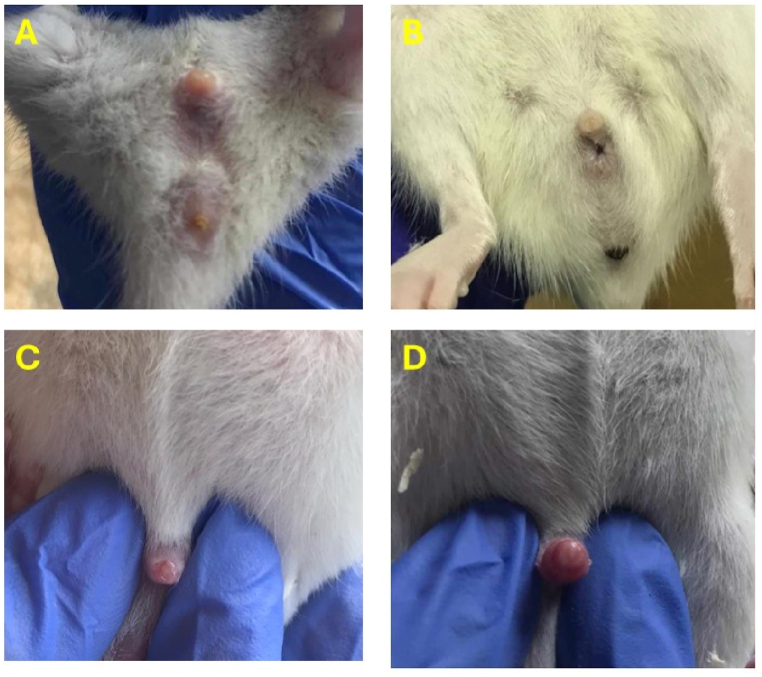
Visual examination of the vagina and preputial separation, where (A) represents the closed vagina, (B) the opened vagina, (C) before preputial separation, and (D) after preputial separation.

For 30 days, the animals were weighed every three other days from 9:00 to 10:00 a.m. Sexual maturation was assessed by recording the vaginal opening [Fig. 2(A and B)] and estrous cyclicity for females, and preputial separation for males [Fig. 2(C and D)]. To analyze the estrous cyclicity, daily vaginal smears were collected between 10 and 11 a.m. using 0.4 mm interdental brushes gently rotated into the vagina lumen. Collected cells were transferred onto glass slides, air-dried, and stained using the Papanicolaou staining process. Specimens were fixed using an alcohol-based solution, followed by nuclei coloration with hematoxylin, cytoplasm coloration with orange G solution, and cytoplasmic components with an eosin-based solution. After dehydration, the smears were examined microscopically. To identify the four estrous cycle stages: proestrus, estrus, metestrus, and diestrus [33,34].

### 2.5 Sample and tissue collection and processing

Biological samples (blood) were collected from the retro-orbital sinus on day 0 and day 30 of the experiment. After blood centrifugation at 4000 rpm for 15 min, serum was collected and stored at −80 °C until ELISA analyses were performed. On day 30, the rats were sacrificed via aesthetic overdose, and genital organs (the ovaries, uterus, vagina for female rats, and the testis, epididymis, and prostate for males) were excised. The right ovary and right testis were homogenized (100 mg sample/2 mL phosphate-buffered saline solution) using a tissue homogenizer (Omni Elite 2.0 with Bead Ruptor Cryo Cooling Unit). After homogenization (6 m/s, 30 s), the homogenate was centrifuged (15,000 rpm; 4 °C; 15 min) and the supernatant was collected and stored at −80 °C until further analyses. The left ovary with its ipsilateral uterine horn and vagina for female rats and the left testis with its epididymis and the prostate were prepared for histopathological analysis.

### 2.6 Histopathological analysis

Histopathological analysis was performed on 40 subjects: 10 rats (5 females and 5 males)/group: CTRL, MP, PC, and LED. The genital organs were fixed in 10 % formalin solution and processed with alcohol (80 %, 95 %, and 100 %) for 60 min, xylene for 60 min, and paraffin for 120 min. Tissues were sectioned (4 μm) and stained with hematoxylin-eosin reagents using the following protocol: xylene soaking for 9 min, alcohol (100 %, 95 %, and 80 %) immersion for 3 min, distilled water rinsing for 4 min, hematoxylin immersion for 1 min, rinsing under running distilled water, and eosin immersion for 1 min. The staining process was stopped by rinsing under running distilled water. Following, the dehydration process was performed by alcohol (80 %, 95 %, and 100 %) slide immersion, and 4 xylene baths. Finally, the mounting step used Mounting solution [35]. The image collection and slide examination were performed using a digital Leica DM750 microscopy system (Leica Biosystems, IL, United States) built-in with an ICC50 digital camera and a LAS EZ operating program. Microscopic lesions (edema and inflammation) were graded using the following numerical scale: 0 – absent; 1- present, mild; 2 – present, moderate and 3 – present, severe.

### 2.7 Cortisol and inflammatory markers assessment

The serum levels of cortisol, inflammatory cytokines TNF-α and IL-6, and tissue levels of matrix metalloproteinases MMP-2 and MMP-9 from ovaries and testis were performed using ELISA technique (Biotek Microplate 50 TS washer and the 800 TS reader, Agilent Technologies Inc., Santa Clara, CA, USA). For cortisol, TNF-α, and IL-6 the detection was performed using the commercially available kits from ElabScience (Houston, Texas, USA), while MMP-2 and MMP-9 in the tissue homogenate were performed using the commercially available kits from Booster Biological Technology (Valley Ave, Pleasanton, CA).

### 2.8 Oxidative stress analysis

The oxidative stress process was evaluated considering the levels of nitric oxide (NO) [36], malondialdehyde (MDA) [37]**,** and total oxidative stress (TOS) [38]. All absorbances were measured using a Jasco V-350 UV–VIS spectrophotometer (Jasco International Co., Ltd., Tokyo, Japan).

### 2.9 Elevated plus maze anxiety assessments

The elevated Plus Maze test, a standardized method for anxiety level evaluation in rodent experimental models, was used to evaluate potential behavioral alteration induced by blue light exposure [39]. The rats were allowed to explore the maze for 4 min in total, and their behavior was recorded (Hikvision camera, Sibiu, Romania) and analyzed based on time spent in open/closed arms and the number of crossings [39]. The measurements were performed on day 29 of the experiment.

### 2.10 Statistical analysis

The program IBM SPSS Statistics (Trial version, IBM, New York, Armonk, USA) was utilized to evaluate the statistical significance of the results obtained from both the biomarkers assessment, as well as the behavioral test between the experimental groups. The Shapiro–Wilk test was employed to assess the normality of data distribution. Data with normal distribution were expressed as Mean ± Standard Deviation (SD), whereas non-normally distributed data were reported as medians with interquartile ranges (25th–75th percentiles). An independent samples *t*-test was applied for comparisons between two independent groups, while one-way ANOVA for more than two groups in normally distributed samples. For non-normally distributed data, group comparisons were performed using the Kruskal–Wallis test. Statistical significance was set at p < 0.05.

### 2.11 Mathematical modeling

The program Matlab® (R2024a Release) was utilized to mathematically model the obtained data. Three input variables were used as follows: Group (CTRL, MP, PC, or LED), Type (Ovary or Testicle), and Sex (Female or Male). A nonlinear function was created to describe the relationship between inputs and outputs. Such a nonlinear model was fitted by adjusting the model parameters using both MMP2 and MMP9 datasets. The model validation was realized by comparison with experimental data using the performance measures: Mean Squared Error (MSE), Root Mean Squared Error (RMSE), and p value. These two models can be used to predict evolutions or to evaluate the MMP-2 and MMP-9 of new input sets.

## 3 Results

### 3.1 General observations and timing of puberty

During the experiments, the observation of animals did not reveal any abnormal signs regarding their general appearance and mortality. All rats displayed normal activities. After 30 days of blue light exposure, both female and male rats presented significantly lower body weight for the rats included in the PC group in the case of females and for the rats included in the PC and LED groups in the case of males (data not shown).

**Fig. 3:**
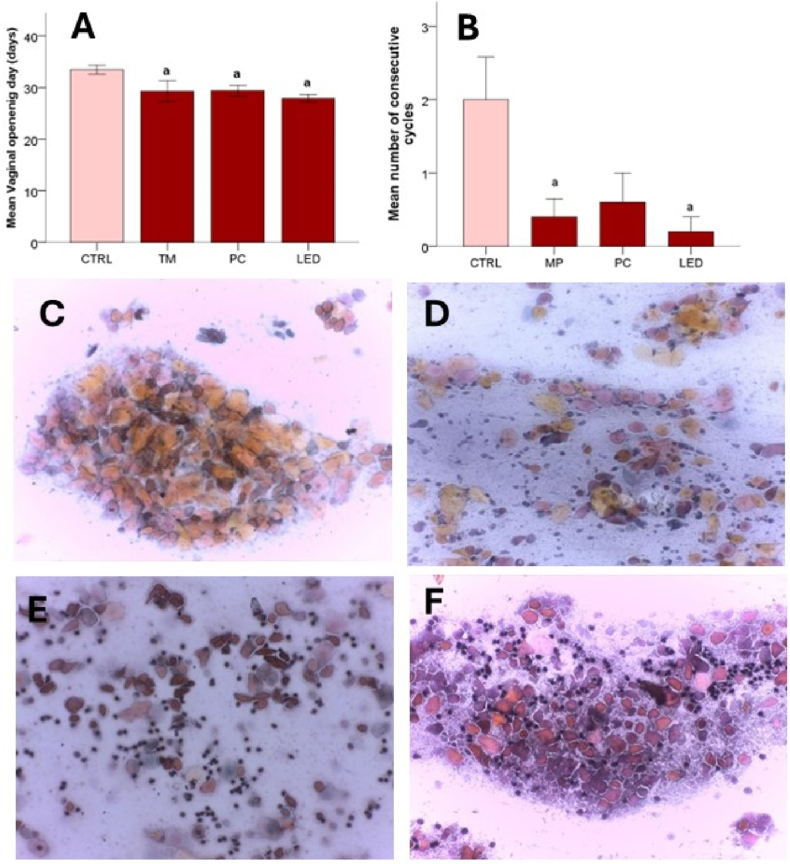
Timing of puberty in female rats where (A) represents the mean vaginal opening day, (B) the number of consecutive cycles (succession of proestrus, estrus, metestrus, and diestrus) where (C) represents the proestrus stage with densely packed conglomerates of squamous cells with small, round nuclei, with a high nucleus to cytoplasm ratio, (D) estrus stage comprising mostly anucleated squames with various irregular shapes (folded or wrinkled) with a diffuse distribution, some rare ghost nuclei and keratin bars between them (E) metestrus stage characterized by the presence of polymorphonucleated neutrophils between the estrus specific squames, and (F) diestrus stage characterized through the large variation in cellular types, sizes, shape and morphology like neutrophils, followed by nucleated squamous cells and sparse anucleated squamous cells.

The installation of precocious puberty was first observed following the vaginal opening (for females), at (33.4 ± 1.33) PND in the case of the CTRL group and significantly earlier for the other groups (29.27 ± 3.34 PND for MP – p < 0.003; 29.40 ± 1.58 PND for PC- p < 0.001; 27.90 ± 1.19 PND for LED - p < 0.001) (Fig. 3A). The preputial separation appeared at 48.20 ± 1.30 PND among individuals included in the CTRL group and was statistically significant among groups. In the male rats exposed to blue light, preputial separation occurred significantly (p < 0.001) earlier (43.20 ± 0.83 PND for MP, 39.80 ± 1.64 PND for PC, and 37.80 ± 1.09 PND for LED).

The number of consecutive estrous cycles (Fig. 3B) was assessed following the succession of the four stages: proestrus (Fig. 3C), estrus (Fig. 3D), metestrus (Fig. 3E), and diestrus (Fig. 3F). The succession of cycles was registered considering that proestrus with an average duration of 14h and metestrus with an average duration of 6–8h could have been missed due to their short duration. Estrus with an average duration of 24–48h and diestrus with an average duration of 48–72h were expected to be present. Thus, the mean number of consecutive cycles was significantly lower for the female rats exposed to the blue light from MP (p < 0.028), and LED (p < 0.014)(Fig. 3B).

### 3.2 Effect of blue light exposure on serum proinflammatory cytokines

**Fig. 4:**
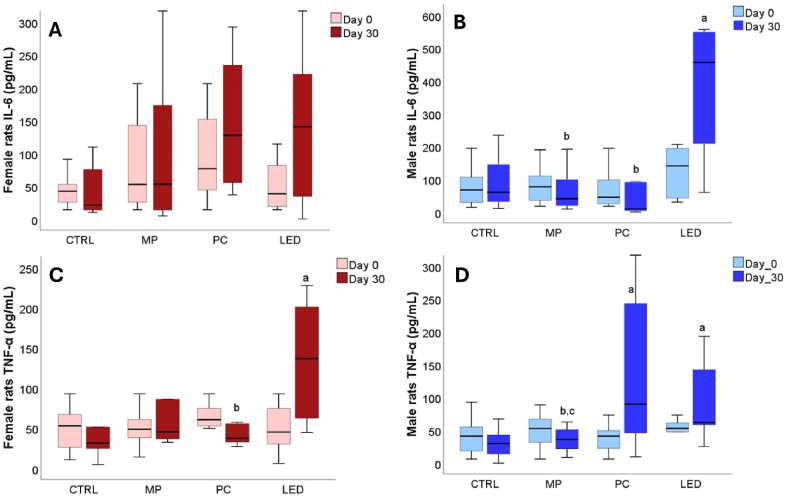
Comparison of female and male serum levels of pro-inflammatory cytokines IL-6 (A and B) and TNF-α (C and D). Animals of both sexes were grouped according to the light exposure sources in CTRL (=no exposure), MP (mobile phone), PC (computer screen), and LED (blue light LED). The variation between proinflammatory cytokines is represented using boxplots where the midline accounts for the median values, the extreme lines indicate the first and third quartile values, where a had p < 0.05, versus CTRL (day 30), b had p < 0.05, versus LED (day 30); c had p < 0.05, versus PC (day 30).

At PND 21 days, considered day 0 of the experiment, the proinflammatory markers (IL-6 and TNF-α) serum values were not statistically different among the different female or male groups [Fig. 4A-D]. After 30 days of blue light exposure, the group exposed to the blue LED light source presented significantly higher serum levels of IL-6 (p = 0.004) in the case of males (Fig. 4B), and TNF-α (p = 0.003 and p = 0.006) for female and male groups [Fig. 4C, D], respectively as compared to the CTRL group. Significantly higher TNF-α (p = 0.007) differences when compared to the CTRL group were observed for male rats after 30 days of PC light exposure as well (Fig. 4D). Higher levels of TNF-α were also observed for females exposed to mobile phone MP light but with no statistical significance.

### 3.3 Effect of blue light exposure on tissue matrix metalloproteinases

**Fig. 5:**
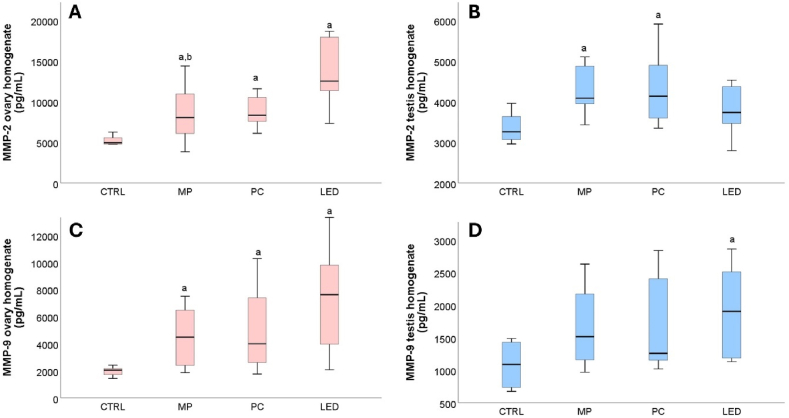
Comparison of female and male tissue levels of matrix metalloproteinase 2 (MMP-2) (A and B) and matrix metalloproteinase 9 (MMP-9) (C and D). Animals of both sexes were grouped according to the light exposure sources in CTRL (=no exposure), MP (mobile phone), PC (computer screen), and LED (blue light LED). The variation between metalloproteinases is represented using boxplots where the midline accounts for the median values, the extreme lines indicate the first and third quartile values, where a had p < 0.05, versus CTRL (day 30), and b had p < 0.05, versus LED (day 30).

After 30 days of controlled blue light exposure, ovary tissue levels of both MMP-2 and MMP-9 varied significantly among female rat groups [Fig. 5A, C]. Higher values were observed in all exposed groups (p = 0.006 and p = 0.013 for the PC group, and p = 0.046 and p = 0.025 for the MP group) with particularly elevated levels in females exposed to LED blue light (p = 0.001, respectively p = 0.002). In the case of male rats, testis tissues showed significantly higher levels of MMP-2 in the MP (p = 0.002) and PC (p = 0.008) groups as compared to the CTRL group (Fig. 5B). The MMP-9 were significantly higher levels only in the LED group (p < 0.031) when compared to the CTRL group (Fig. 5D).

### 3.4 Effect of blue light exposure on serum oxidative stress parameters

**Fig. 6:**
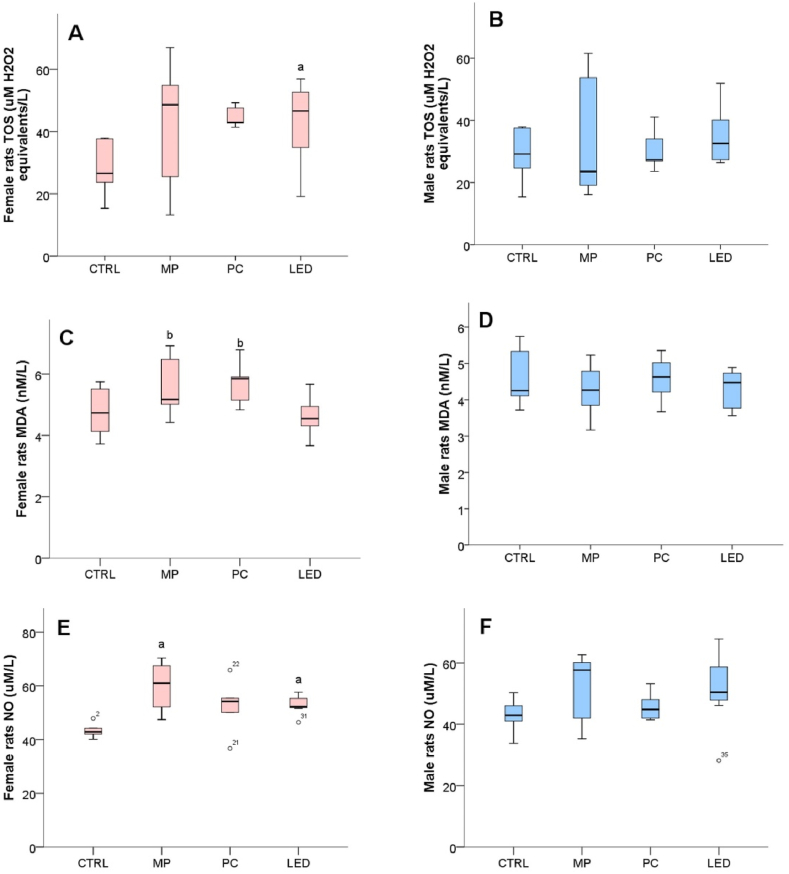
Comparison of female and male serum levels of Total Oxidative Stress (TOS) (A and B), malondialdehyde (MDA) (C and D), and nitric oxide (NO) (E and F). Animals of both sexes were grouped according to the light exposure sources in CTRL (=no exposure), MP (mobile phone), PC (Computer screen), and LED (blue light LED). The variation between oxidative stress parameters is represented using boxplots where the midline accounts for the median values, the extreme lines indicating the first and third quartile values; a had p < 0.05, versus CTRL (day 30), b had p < 0.05, versus LED (day 30).

The effect of blue light exposure on the investigated oxidative stress parameters was gender-specific [Fig. 6A-F]. While male rats did not show any statistical difference between the different groups [Fig. 6B, D, F], serum levels of TOS and NO were significantly influenced in the case of female rats [Fig. 6A, E]. Total oxidative status was significantly increased in female rats after 30 days of exposure to LED light (p = 0.05) vs CTRL group. Malondialdehyde in female rats from the MP and PC groups showed significantly higher levels than rats included in the LED group (p = 0.05 and p = 0.033, respectively), but without significance when compared to CTRL group rats (Fig. 6C). Nitric oxide was significantly increased after MP light exposure (p = 0.001) and LED light exposure (p = 0.025) vs CTRL (Fig. 6E).

Pearson test between the investigated oxidative markers in female rats showed a significant correlation of MDA and NO (p < 0.05), TOS and NO (p < 0.01), and MDA and TOS (p < 0.01). In male rats, the correlation was present only for TOS and NO (p < 0.05).

### 3.5 Histopathological assessment

In the case of males, moderate interstitial edema was found in the stroma of the testis, epididymis, and prostate in all male subjects. Moreover, in the MP and PC groups, we identified congestion and red blood cell extravasation in the superficial, subcapsular stroma. Mild chronic inflammation (inflammatory infiltrates composed of lymphocytes and plasma cells) was identified in the stroma of the prostate of subjects belonging to the LED group, and in the stroma of the epididymis in the PC and LED groups. However, there was no statistical significance regarding these alterations within the four groups. From a microscopic point of view, spermatogenesis was unaffected, as cells belonging to the germinative epithelium (spermatogonia, spermatocytes, spermatids, and spermatozoa) were all present and showed a correct histological distribution within the thickness of the epithelium. The seminiferous epithelium had a homogenous height among tubules and germ cells showed no structural, nor cytologic atypia. Leydig cells were also identified in the intralobular stroma and showed no microscopic changes.

**Fig. 7:**
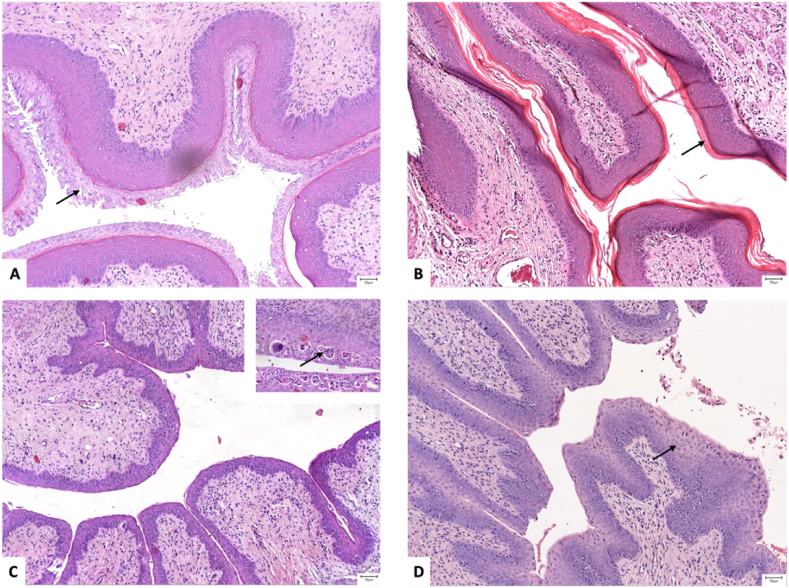
Aspects of the vaginal mucosa in different stages of the estrus cycle: **A.** Proestrus - an evident stratum mucification is present at the superficial layer of the surface epithelium (arrow); **B.** Estrus – the epithelium presents a well-developed stratum corneum (arrow) and no stratum mucification; **C.** Metestrus– the epithelium has a thin germinal layer and lacks stratum mucification and stratum corneum; frequent intraepithelial microabscesses were also identified (arrow, inset) and **D.** Diestrus – plump squamous cells (arrow) were present in both the germinal and spinous layers (A-D: Hematoxilin and Eosin, 10x).

Regarding the females, for each subject, the stage of the estrous cycle was assessed within three different structures: 1) the vaginal epithelium [Fig. 7A-D], 2) the endometrium, and 3) the luteal bodies of the ovaries.

There was a significant (p < 0.001), strong positive correlation between the estrous stages in the vagina and endometrium, and also a positive significant mild correlation between the vagina estrous stage and the stage observed in the luteal bodies (p < 0.019).

**Fig. 8:**
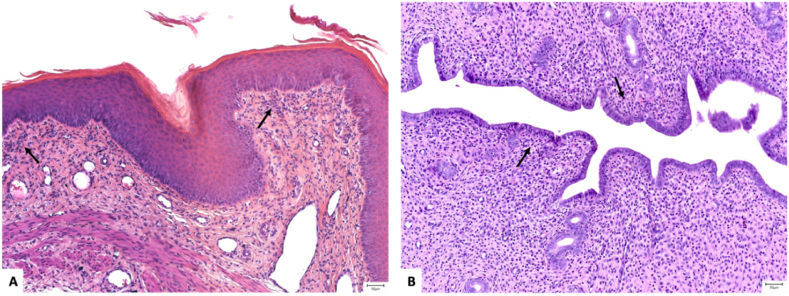
Moderate amounts of chronic, lymphocytic inflammation (arrows): **A.** in the vaginal stroma, with enhancement in the superficial and perivascular areas, and **B.** in the endometrial stroma (A, B: Hematoxilin and Eosin, 10x).

While control subjects presented a minimum amount of acute inflammatory cells (polymorphonucleated neutrophils and eosinophils) in the uterine and vaginal stroma, female subjects belonging to all groups exposed to blue light demonstrated chronic inflammation [Fig. 8A, B]. Mild or moderated amounts of lymphocytes were identified in the connective tissue of the endometrium and vagina of the great majority of female subjects; there was no case of marked inflammation. Accordingly, a significant statistical difference regarding vaginal inflammation was observed between subjects included in the CTRL group and the ones exposed to the blue light coming from MP (p < 0.024), PC (p < 0.001), and LED (p < 0.001), respectively. The differences in the endometrial inflammation within groups did not reach statistical significance.

Female subjects from the study groups also presented more frequent luteal bodies in the histopathological sections of the ovaries. The subjects from the control group, had an average of luteal bodies/ovary of 2.5 ± 1.73, while the averages of luteal bodies/ovary within the MP, PC, and LED groups were 5.4 ± 1.67; 4.4 ± 1 and 4 ± 3.6, respectively. Only female rats in the MP group had a significantly higher number of luteal bodies/ovaries compared to the CTRL group (p = 0.038).

### 3.6 Mathematical modeling of evaluated tissue parameters and histological data

For the MMP-2 dataset, the best performance measures are obtained with the model

Y = 0.2472 × (pi / 2 + atan((x1) + 0.7063./ (−0.9587)) – 0.2242 x2 – 0.2236x3 – 0.1429x4 + 0.1004x5 - 0.1445x6 + 0.1004x7 – 0.1445x8

where: x1, x2, x3, ×4 – Group 1, 2, 3, or 4.

x5 – Ovary, ×6 – Testicle

x7 – Female, ×8 – Male.

The performance measures are: MSE = 0.0318, RMSE = 0.1783, P-value = 4.2960e-13.

For the MMP-9 dataset, the best performance measures are obtained with the model

Y = 0.2462 × (pi / 2 + atan((x1) - 0.2258./ (−1.0824)) – 0.1081x2 – 0.0689x3 + 0.0201x4 + 0.0340x5 - 0.1136x6 + 0.0339x7 – 0.1136x8

where the same variables are used as in the previous case.

The performance measures are: MSE = 0.0243, RMSE = 0.1560, P-value = 4.368e-09.

**Fig. 9:**
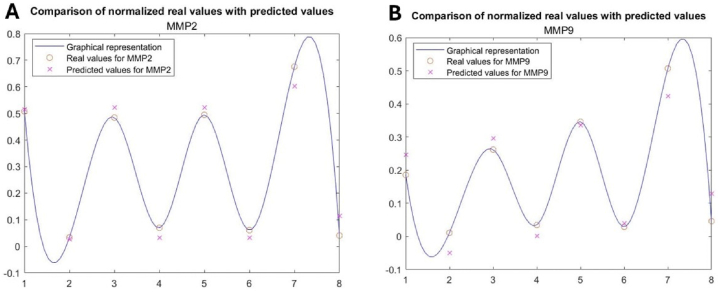
Comparison between experimental data and model-predicted values: **A**) for the MMP-2 dataset; **B**) for the MMP-9 dataset.

The results obtained with these two models are plotted in [Fig. 9A-B].

### 3.7 Effect of blue light exposure on cortisol stress response

**Fig. 10:**
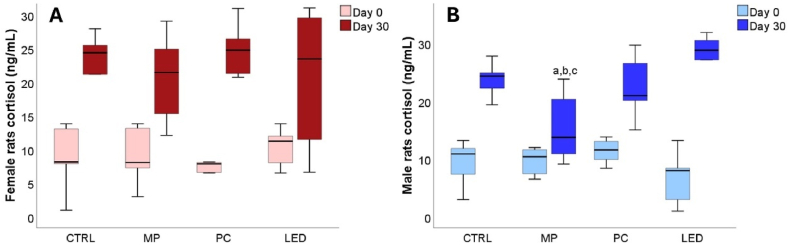
Comparison of female and male serum levels of cortisol (A and B). Animals of both sexes were grouped according to the light exposure sources in CTRL (=no exposure), MP (mobile phone), PC (computer screen), and LED (blue light LED). The variation between cortisol is represented using boxplots where the midline accounts for the median values, the extreme lines indicating the first and third quartile values where a had p < 0.05, versus CTRL (day 30), b had p < 0.05, versus LED (day 30); c had p < 0.05, versus PC (day 30).

The secreted cortisol levels in rats' plasma were detected to illustrate the rat's response to the blue light-induced stress. As compared to day zero, after 30 days of controlled exposure to blue light, cortisol was physiologically increased in all groups [Fig. 10A, B]. Increased levels of cortisol were detected only for the male rats exposed to LED blue light (p = 0.01). Interestingly, the cortisol levels detected in the group exposed to mobile phone MP blue light were significantly lower (p = 0.003) than all the groups included in the study (Fig. 10B).

### 3.8 Effect of blue light on anxiety behavior in rats

**Fig. 11:**
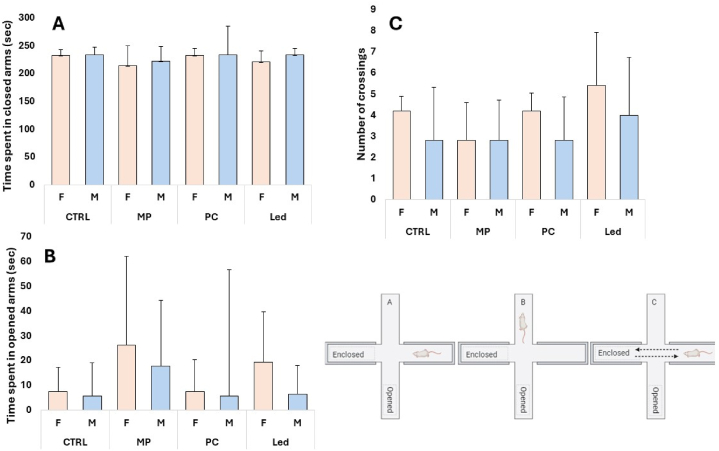
Rat female and male time spent in the closed arms (A), opened arms (B), and the number of crossings (C) between the closed arms of the elevated plus maze.

Tested over a single session, female and male rats exhibited a similar profile of low open-arm exploration, similar to those in the CTRL groups. No statistical difference was detected among groups [Fig. 11A-C], highlighting that the exposure to blue light did not affect their anxiety level.

## 4 Discussions

This study revealed that exposure to blue light from various sources (MP, PC, and LED) affected the onset of puberty in female and male rats. Precocious puberty occurred earlier in the case of females as observed following the vaginal opening day and reduced consecutive cycles number. In the case of males, precocious puberty was evaluated following earlier preputial separation. Vaginal opening in female rats and preputial separation in male rats are considered signs of precocious puberty [3,40,41]. In normal physiological conditions, the vaginal opening occurs between PND 32 and 34 [42], while the preputial separation between PND 33 to 55 [40]. Regarding the estrous cycles, the regular ones are defined as a 4 or 5-day cycle (with 2 days of estrus and 2 or 3 consecutive days of diestrus), while the irregular ones are defined as 6 or 7-day cycles with persistent estrus or diestrus [43]. Since the onset of vaginal opening day and the number of consecutive regular estrous cycles in females, together with preputial separation in males were significantly decreased in the rats exposed to blue light we could sustain the hypothesis by which prolonged blue light exposure can induce precocious puberty. In line with our study, several other *in vivo* animal studies reported that exposure to constant light accelerates sexual maturation [8,[43], [44], [45]].

The incidence of precocious puberty is on the rise among children worldwide [5,46]. The reasons behind the increased occurrence of precocious puberty onset in these children remain unclear. Most studies indicate that premature activation of the hypothalamic-pituitary-gonadal (HPG) axis leads to gonadotropin-dependent precocious puberty, characterized by the early initiation of puberty driven by hormones from this axis [47]. However, the processes that trigger HPG activation are intricate, and in many cases, the exact cause of this early activation is not well understood. Still, risk factors like wrong dietary habits (e.g. consumption of sugary snacks, soft drinks, and fried foods), a sedentary lifestyle, affected sleep patterns (including delayed or disrupted sleep), and excessive screen time exposure [48] were linked with precocious puberty onset [9,49]. Therefore, the consequences associated with these risk factors can logically include adrenal activation, physical and psychological stress, excessive fatty tissue accumulation, inflammation, and oxidative stress enablement as identified in recent research [[50], [51], [52]]. Among the processes involved in the induction of precocious puberty, inflammation, and oxidative stress remain insufficiently investigated. To our knowledge, this is the first study aiming to explore the role of inflammation and oxidative stress in induced precocious puberty by blue light exposure.

Proinflammatory biomarkers, such as IL-6 and TNF-α, are recognized for their potential role in the development of pubertal conditions [53]. Our study demonstrated that after 30 days of exposure to blue light, both female and male rats exhibited significantly elevated serum levels of TNF-α compared to the control group, and higher IL-6 levels in the case of male rats. Notably, the LED-emitted light impacted both sexes, whereas the PC-emitted light affected only male rats. Other studies also identified that persistent blue light (24 h monochromatic blue light at 450 nm) in mice fed with a high-fat diet affected kidneys' inflammatory status and activated the pro-apoptosis pathway through the induction of the relative mRNA levels of TNF-α, Il-6, Il-1β, and Mcp-1 [54]. IL-6 and TNF-α are frequently cited in the literature regarding the effects of psychological factors on immune response [[55], [56], [57]]. IL-6 is synthesized by leukocytes as well as hepatic and adipose tissues, and it influences various systems, including cardiac, pulmonary, excretory, digestive, nervous, and skeletal systems. In addition to its role in inflammation, IL-6 stimulates C-reactive protein (CRP) production in hepatocytes and facilitates the transition from acute to chronic inflammation [58]. TNF-α is crucial for immediate inflammatory responses, with its levels returning to baseline within hours. It is functionally recognized to initiate a cascade of various inflammatory molecules, encompassing a range of cytokines and chemokines such as IL-6, Interleukin-1 beta (IL-1β), Interleukin-8 (IL-8), Interleukin-12 (IL-12), Interleukin-17 (IL-17) and others [59]. In addition to its pivotal role in initiating and amplifying the inflammatory cascade, TNF-α is involved in modulating oxidative stress, recruiting immune cells and adhesion molecules, inducing apoptosis, and facilitating healing and tissue-specific repair mechanisms [59]. High levels of TNF-α can also disrupt homeostasis by affecting circadian rhythms, appetite, and sleep [60]. Alongside IL-6, TNF-α can interfere with the regular functioning of the hypothalamic-pituitary-adrenal (HPA) and hypothalamic-pituitary-gonadal (HPG) axes [61,62]. Our findings align with previous research highlighting the role of IL-6 and TNF-α in stress-related immune responses. Elevated levels of these biomarkers in response to blue light exposure suggest potential mechanisms through which environmental stressors can influence pubertal development and immune function. Further research is needed to explore the differential effects of various light sources and to understand the underlying mechanisms contributing to gender-specific responses.

Further, tissue MMP-2 and MMP-9 were tested in the ovary and testis tissues. It was observed that both females and males presented statistical differences among groups after 30 days of exposure to the blue light. In the case of female rats, significantly higher levels were observed for both MMP-2 and MMP-9, regardless of the type of emitting device. In the case of male rats MMP-2 levels were significantly increased after exposure to MP and PC light whereas MMP-9 levels were significantly increased after exposure to LED light.

Tissular growth, remodeling, repair, and morphogenesis are crucial aspects of pubertal development [63]. The morphogenesis of the extracellular matrix is age-dependent, which calls for specific patterns of MMPs to allow for rapid tissue growth during childhood and tissular expansion and remodeling during puberty [63]. The main action of MMPs is remodeling the extracellular matrix by breaking down its components. Regulating the proteolytic breakdown of the extracellular matrix could be a key mechanism for controlling the availability and activity of growth factors, thereby affecting tissue differentiation during organ development [64]. MMPs also regulate TNF-α and IL-1b levels, making them a regulator of the inflammatory response. Through these two mechanisms of action, MMPs play a role in the evolution of various diseases affecting multiple systems, modulation of different processes on a physiological level, and relating to various pathological pathways [65,66]. In female individuals, ovarian-located MMP-2 has been shown to play a role in follicular rupture as it degrades the extracellular matrix at the apex of the ovary [67]. Further, MMP-2 activity has also been linked to androgen hormone levels as many studies attribute to testosterone the role of MMP regulator [68,69] and the activation of MMP-2 is regulated through steroid hormone secretion [70]. Despite that, there is currently insufficient information on one of the most important proteinase classes, the MMPs, regarding their production, clearance, and concentrations during pediatric developmental stages [63]. The findings of our study suggest that blue light can trigger the overproduction of MMPs for both sexes. The correlations between tissue expression of MMP-2 and MMP-9 were also expressed by mathematical models. These equations can capture the dynamics of MMP-2 and MMP-9 evolutions influenced by group (CTRL group, MP group, PC group, and LED group), type of tissue (ovary or testicle), and gender (female or male). Moreover, these models can be used as predictors for the evolution or evaluation of possible MMP-2 and MMP-9 levels for specific individuals.

The histopathological examination indicated that the ovaries and testis rats did not present tissular changes with statistical differences among groups. An organic alteration would probably be observed after a longer time of exposure to blue light. The presence of interstitial edema, as a pivotal component of inflammation in the stroma of the testis, epididymis, and prostate in almost all male subjects could reflect an adaptative defense mechanism against tissue injury. The same conclusions could be depicted for female rats as well. According to the stage of the rat estrous cycle, the endometrium can present various amounts of immune cells, which thus serves as a normal histologic finding. The literature describes these immune cells as polymorphonucleates neutrophils or eosinophils, which can be identified in high numbers in the first two stages, of preoestrus and estrus [71]. We identified cases with mild and moderate immune infiltrates in the endometrial stroma of 15 out of 15 subjects and the vaginal stroma of 14 of these 15 subjects, but no cases of marked immune infiltrates. Moreover, in the study groups, we reported chronic, lymphocytic inflammation, without infiltration of acute inflammatory cells.

Concerning oxidative stress parameters, it was found that, in rat females, 30 days of exposure to blue light LED significantly increased TOS. Nitric oxide was also significantly increased in females after 30 days of exposure to MP and LED. Male rats were not significantly affected, but the same increasing trend was observed. Our results follow other studies that investigated the influence of prolonged blue light exposure in different experimental settings to see the changes in inflammatory and oxidative stress status processes. Thus, it was reported that long-term exposure of male rats to low light levels during the night affected renal redox, immune redox balance, and the biological clock that regulates the changes in circulating immune cells [72]. Another study found that nighttime low-intensity blue light-induced oxidative stress and inflammation in the hippocampus, which led to memory impairment in mice [73]. It was also reported that high levels of ROS in males, affect the Leydig cells, which are responsible for the maturation of the reproductive system, as well as post-puberty spermatogenesis [74,75]. Due to their proximity to the macrophages located in the testis, Leydig cells can become damaged because of oxidative stress which could lead to hormonal issues that affect the onset of puberty [76]. Also, research on both humans and animals has shown a connection between oxidative stress and anxiety. Findings suggest that oxidative stress may serve as a crucial molecular link between dysfunction in the HPA axis and mental disorders. Additionally, it has been noted that stress-induced elevations in cortisol levels enhance glucose metabolism and lead to an increased production of reactive oxygen species [77].

The impact of blue light on rats' behavior was further analyzed both physiologically (by evaluating cortisol levels) and behaviorally (by evaluating anxiety). Cortisol is the glucocorticoid hormone produced by the HPA axis, responsible for responding to stressful stimuli, as well as regulating many processes [78]. High levels of cortisol have been linked by researchers with major changes affecting both the individual's cognitive functions and behavior [79]. Alongside other hormones, cortisol can also disrupt the normal circadian rhythm and metabolic functions as a response to blue light exposure at night by its effect on sleepiness [80,81]. Our study showed that blue light exposure increased the male rats' levels of cortisol after 30 days of exposure, especially to the LED blue light. Interestingly, the MP blue light significantly decreased cortisol production. The production of cortisol in female rats was not affected. There's a complex circadian rhythm pattern that modulates cortisol levels throughout the day [78]. However, unlike the modifications that occur in melatonin levels, cortisol secretion is much more correlated to the transitions from dark to light and at a lower scale, from light to dark. In humans, cortisol concentration starts gradually increasing upon waking up, reaching its peak 30 min to an hour later. For rats, the elevation and peak in cortisol levels are registered during the evening, as they are nocturnal animals [81]. Thus, we can assume that the decreased levels of cortisol in rats exposed to the blue light emitted by MP induced an alteration in cortisol production probably linked to an altered perception regarding the start of the daily activity period. It is expected that the action of stressful factors, regardless of their nature, triggers subsequent neutral pathways located in the central nervous system, causing the diencephalic centers to commence a behavioral, endocrine, or autonomic response [82]. Further, as the major stress hormone, cortisol can determine behavioral changes [83]. This study did not find altered anxiety levels in rats as evaluated by the elevated plus maze test after 30-day exposure to blue light.

Even though we found that blue light, especially the one emitted by the LED source (concentrated around 450–470 nm), can enhance inflammation, oxidative stress processes, and cortisol levels, histological changes were discrete, and animal behavior was not affected. More studies will also be needed, over a longer time frame period to evaluate the incidence of both physical and psychological conditions during puberty and to be able to correlate them with the developmental changes that are characteristic during this stage. Also, the differences between female and male rats suggest that the mechanisms by which this alteration occurs need further approach.

## 5 Limitations of the study

Due to the complexity of the study, the serum cortisol levels were detected in samples taken in the morning at 11–12 a.m. We could not take the samples in the late evening after 7 p.m. when the rats' peak activity begins.

## 6 Conclusions

This study demonstrates that prolonged blue light exposure from smart devices significantly induces precocious puberty in both male and female rats, accelerating sexual maturation. Blue light exposure increases inflammatory markers (IL-6, TNF-α), matrix metalloproteinases (MMP-2, MMP-9) levels, and oxidative stress markers with distinct gender-specific effects. The study successfully developed a mathematical model to capture the dynamics of MMP-2 and MMP-9 expression across the different groups, tissue types (ovary or testicle), and genders (female or male). The utilization of mathematical modeling can offer an integrative approach to evaluate the biochemical impacts of blue light exposure in reproductive tissues. It may also aid in the assessment and prediction of MMP levels in specific conditions. Further, female rats showed elevated TOS, NO, and MDA levels, whereas male rats had either increased or decreased cortisol levels dependent on the light-emitting source. These findings suggest that environmental exposure to blue light from everyday electronic devices could be a contributing factor to the increasing prevalence of idiopathic pediatric precocious puberty, warranting further investigation that could impact public health interventions.

## CRediT authorship contribution statement

**Raluca Maria Pop:** Writing – original draft, Supervision, Funding acquisition, Conceptualization. **Luciana Mădălina Gherman:** Writing – original draft, Methodology, Funding acquisition, Conceptualization. **Elena-Mihaela Jianu:** Writing – original draft, Funding acquisition, Conceptualization. **Ștefan Horia Roșian:** Methodology, Investigation. **Mădălin Mihai Onofrei:** Methodology, Investigation. **Lavinia Patricia Mocan:** Methodology, Investigation. **Veronica Sanda Chedea:** Writing – review & editing, Validation, Methodology. **Ioana Corina Bocsan:** Validation, Methodology, Investigation. **Dragoș Apostu:** Methodology, Investigation. **Andreea Roxana Todea:** Methodology, Investigation. **Eva Henrietta Dulf:** Validation, Resources. **Jeanine Cruceru:** Methodology, Investigation. **Carmen Mihaela Mihu:** Visualization, Resources. **Alina Elena Pârvu:** Writing – review & editing, Validation, Methodology. **Anca Dana Buzoianu:** Writing – review & editing, Visualization, Validation, Supervision, Resources.

## Ethical statement

This experiment was carried out following institutional ethical standards and approved by the University of Medicine and Pharmacy “Iuliu Hațieganu” Ethics Committee and by the Sanitary-Veterinary and Food Safety Directorate from Cluj-Napoca (authorization number 380/August 25, 2023).

## Funding

This project was supported by funding from the Academy of Romanian Scientists’ Research Project Competition for Young Researchers “AOSR-TEAMS-II” EDITION 2023–2024 – “Digital Transformation in Science” (No 28/April 11, 2023).

## Declaration of competing interest

The authors declare the following financial interests/personal relationships which may be considered as potential competing interests: Pop Raluca Maria reports financial support was provided by 10.13039/501100019895Academy of Romanian Scientists. If there are other authors, they declare that they have no known competing financial interests or personal relationships that could have appeared to influence the work reported in this paper.
